# The role of digital mental health resources in encouraging help-seeking: A qualitative study of university students’ experiences

**DOI:** 10.1017/gmh.2026.10216

**Published:** 2026-05-18

**Authors:** Junming Ma, Joo Siang Tan, Huaipeng Shi, Yuehan Bao

**Affiliations:** 1 Department Educational Studies & Behavioural Sciences, Faculty of Educational Sciences and Technology, Universiti Teknologi Malaysia, Malaysia; 2 Shandong Urban Construction Vocational College, China

**Keywords:** digital mental health, help-seeking, Chinese university students, interpretative phenomenological analysis (IPA), digital fatigue

## Abstract

Digital mental health tools are increasingly available to university students, yet evidence remains limited on how Chinese students engage with these resources and how such engagement shapes help-seeking. This exploratory study examined Chinese postgraduate students’ lived experiences of using digital mental health tools and the factors influencing engagement and perceived value. Semi-structured interviews were conducted with 10 postgraduate students (aged 23–30) who had used mental health apps, AI chatbots, or online counseling services. Data were analyzed using Interpretative Phenomenological Analysis. Four themes emerged: accessibility and anonymity lowered barriers to help-seeking; digital tools supported self-regulation but were viewed as complements rather than substitutes for professional care; sustained engagement was limited by impersonal responses, usability issues, credibility concerns, and digital fatigue; and stigma, self-reliance, and peer norms shaped willingness to seek support. Participants also highlighted concerns about safety and escalation pathways. Overall, digital tools may provide timely, low-threshold support, but their impact may be improved through stronger personalization and governance and through blended models that integrate peer and professional care with timely escalation. Given the small, context-specific sample, these results are exploratory and intended to generate hypotheses rather than provide generalizable conclusions.

## Impact statement

This study provides timely insights into how digital mental health resources are shaping help-seeking among university students, particularly within highly digitized contexts. By focusing on lived experiences, it moves beyond questions of “whether digital tools work” to examine how, when and why students actually engage with them. The findings highlight that digital platforms can play a critical role in lowering barriers to initial help-seeking especially through anonymity, convenience and 24/7 availability making them particularly valuable for students who may otherwise avoid formal support due to stigma or access constraints.

At the same time, the study identifies important limitations that have direct implications for policy, practice and design. Students do not view digital tools as replacements for professional care, but rather as supplementary supports. Issues such as lack of personalization, digital fatigue and concerns about credibility and safety can reduce sustained engagement. These insights underscore the need for more user-centered, culturally sensitive and ethically governed digital mental health systems.

The wider impact of this research lies in informing universities, mental health practitioners and technology developers about how to better integrate digital tools into existing support structures. Specifically, the findings support the development of “blended care” models that combine digital access with human support and clear escalation pathways for risk. Additionally, the study contributes to ongoing debates on the ethical use of AI in mental health by emphasizing accountability, transparency and user trust.

Overall, this research offers practical guidance for improving digital mental health strategies in higher education and contributes to a more nuanced understanding of student help-seeking in the digital age.

## Introduction

The growing reliance on digital mental health resources among university students reflects a major shift in how mental health support is delivered in higher education worldwide. Across settings, university students face multiple stressors – including academic demands, social pressures and the transition to adulthood – which increase vulnerability to anxiety, stress and depression. At the same time, many students continue to encounter barriers to traditional, in-person services, including stigma, long waiting times and persistent shortages of mental health professionals (Priestley et al., 2021; Zhang, 2024). In response, digital mental health interventions such as mobile apps, artificial intelligence (AI)-based support and online therapy services have grown quickly. They are increasingly seen as a practical way to improve access to care for university students (Dallinger et al., 2022). Within this evolving landscape, online mental health resources are more convenient and less time-consuming than in-person therapy. They also offer some level of anonymity and convenience, making it easier for people to ask for help without fear of judgment – which can be a serious pitfall of traditional, face-to-face interactions. Further, this kind of digital service overcomes barriers to mental health support such as geography, waiting lists and financial expense, issues that disproportionately affect university students (Balcombe and De Leo, 2021). Mobile apps, virtual counseling and AI-driven chatbots are particularly appealing because they offer students support on their own terms, anywhere, 24/7, independent of regular therapy sessions. This convenience factor is particularly relevant in a university environment, where students may have irregular timetables and may perceive conventional mental health care as time-consuming or disruptive (Torous et al., 2021).

Within this broader international trend, similar patterns are evident in China, where mental health needs among university students are substantial; Ning et al. (2024) reported a high prevalence of mental disorders among Chinese students, with estimates suggesting that up to 30% may experience mental illness. In the Chinese university, student mental health support is typically organized by campus counseling centers, with increasing incorporation of tele components such as hotline-based services and platform-delivered resources. During and after the COVID-19 period, multiple forms of online mental health support were rapidly implemented and scaled, reflecting a broader policy and institutional push to expand access to psychological support via digital channels (Liu et al., 2020; State Council of the People’s Republic of China, 2022). China’s digital mental health ecosystem includes online counseling and therapy apps such as Yi Xinli, JianDanXinLi, HaoXinQing and Zhaoyang Doctor, alongside AI- and self-guided tools including Woebot-type Cognitive Behavioral Therapy (CBT) chatbots and mindfulness apps such as ChaoXi, Now Meditation and Heartly Lab. In parallel, universities are increasingly integrating AI-based assessment, big-data early warning systems and virtual reality (VR)-assisted relaxation training into campus mental health services (Chen, 2025), while hospital-led internet psychiatry services provide online follow-ups, e-prescriptions and continuing care, particularly for mild-to-moderate conditions. In practice, however, universities may differ in how digital services are implemented and connected to in-person care, including differences in service capacity, integration arrangements and the clarity of referral and crisis response pathways (Yu et al., 2025).

But even as digital mental health platforms become more popular, we still have much to learn about their effects on student help-seeking behavior. Although the efficacy of digital interventions in symptom reduction for mental health disorders has been demonstrated in numerous studies (Harrer et al., 2018), the impact of digital platforms on help-seeking attitudes among students has not been as well investigated (Löchner et al., 2025). The vast majority of students remain reluctant to use online mental health tools, as they consider such tools as ineffective and too impersonal (Pretorius et al., 2019). Further, digital interventions may provide immediate fulfillment in accessing resources, but may not potentially address the emotional needs for more complex mental health problems that users could have (Rony et al., 2024; American Psychological Association [APA], 2025).

Although digital mental health resources offer clear benefits, their actual utilization use is strongly shaped by two factors: psychological readiness and digital fatigue. In this study, psychological readiness refers to an individual’s emotional preparedness and willingness to recognize and disclose mental health difficulties, which facilitates engagement with support. By contrast, digital fatigue refers to cognitive and motivational depletion resulting from sustained technology use, which can hinder engagement and contribute to dropout. However, access to digital mental health tools extends beyond university services and is available across multiple platforms. Hence, this study focuses on university students’ overall experiences of accessing and engaging with digital mental health support. Existing research has largely focused on whether digital tools work, but we still know little about how university students balance being ready to seek help with feeling worn out by constant digital use. In this study, we used Interpretative Phenomenological Analysis (IPA) to examine how the interaction between students’ psychological readiness and digital fatigue shapes help-seeking, with attention to the sociocultural context in which these experiences occur.

### Theoretical frameworks

Two theoretical models that may contribute to our ability to understand how such digital mental health resources may be embraced by university students include the Technology Acceptance Model (TAM) and Self-Determination Theory (SDT).

The TAM Developed by Davis (1989), intended to explicate the adoption of new technology. TAM suggests that perceived usefulness (PU) and perceived ease of use (PEOU) are the two influential determinants of adoption of technology. In the context of digital mental health tools, PU is the extent to which a student perceives the system will help improve his or her well-being, and PEOU refers to the student’s belief that it is easy to use and to navigate the system. The literature demonstrates that more students would use digital platforms for mental health help-seeking if they believe the platforms are useful and easy to use (Sawrikar and Mote, 2022).

Meanwhile, SDT (Deci and Ryan, 1985, 2000; Ryan and Deci, 2017) holds that motivation is underpinned by three basic psychological needs: autonomy, competence and relatedness. In the digital mental health domain, autonomy involves the degree to which students experience a sense of volition for selecting how and when to use these tools, competence involves their sense of being able to exercise control over their mental well-being through the use of these resources and relatedness refers to the perceived connections students have with the resources or the people behind the resources. Students’ motivation to use the platforms regularly could derive from the extent to which such needs are satisfied on digital platforms.

TAM and SDT are both used as theoretical approaches to the understanding of the determinants of students’ use of digital mental health tools and the help-seeking behavior.

## Literature review

### Barriers to engagement with digital mental health resources

Despite the benefits of digital mental health interventions, there remain barriers to their potential uptake and engagement by university students (Borghouts et al., 2021). The most obvious downside is absence of personal touch, human interactions (Madrid-Cagigal et al., 2025). Although many digital platforms are quick and convenient, they lack the emotional support of traditional face-to-face therapy. Students may feel disconnected and feel like their lifestyle is not understood, especially when they are dealing with complex emotional or psychological struggles (Haque and Rubya, 2022). Though AI-based tools like chatbots are successful in some cases, reaching a level of accurately assessing mental health need of the user and suggesting best solution is not always feasible as well (Thakkar et al., 2024). That could lead users to question whether they really can help and whether they should rely on them as a source of continued mental health care.

Psychological resistance to utilization of care is another significant barrier. There is still remaining stigma hindering mental health help-seeking among students and digital mental health resources might not be adequate to break this social barrier (Gulliver et al., 2010). Even though online students have the opportunity to remain anonymous, there may still remain a sense by which students feel exposed or embarrassed to seek mental health assistance, particularly when they perceive asking for help to be a weakness or inability to fend for themselves (Hammarlund et al., 2018). Furthermore, students may express a belief in self-resilience, a sense that they “ought to be able” to manage their own mental health, also serving to disincentivize the searching for support, including in digital contexts (Mesman et al., 2021).

### The role of social and psychological influences

Social and psychological factors also significantly influence help-seeking among university students. Social norms, family expectations and cultural norms towards mental health also shape whether individuals are more or less supportive of digital mental health tools (Kirkbride et al., 2024). Students who attend schools where mental health difficulties are both discussed openly and normalized are more likely to use online resources for mental health issues. But in more stigmatized social climates, students may be less willing to ask out of fear that their peers will judge or minimize them (Yablon, 2012; Earnshaw et al., 2018). This is particularly the case in universities, where social comparison and peer validation might have a strong impact on students’ attitudes and behaviors regarding mental health care (Lee et al., 2023).

In addition, the psychological readiness is very important to make students be ready to use the digital mental resources. Individuals who perceive themselves as emotionally sturdy or independent may be less likely to utilize available supports even if these supports are accessible (Horgan and Sweeney, 2009; Lattie et al., 2022). Conversely, those students who have previously been exposed to mental health promotion or have an increased awareness of the importance of seeking help may be more open to engaging with such resources (Shek et al., 2024).

Studies previously covered key concerns pertaining to access to and satisfaction with digital mental health services (Bunyi et al., 2021; Alshaikhi et al., 2025); however, there is limited literature that goes into detail regarding the social–psychological–technological dimension of university students and their attitudes toward help-seeking. This qualitative study seeks to fill this gap by focusing on the lived experiences of students engaging with digital mental health services. Based on interpretivist phenomenological analysis, the research aims to investigate the underlying factors on the willingness of students for help-seeking in psychological, social and technological perspectives. It will also identify student-level barriers and facilitators related to their engagement with such resources that can inform the development of more responsive and user-friendly digital mental health interventions.

## Methodology

In the current study, lived experiences of a support seeking process from a digital mental health perspective were examined among university students through considering perceived accessibility, effectiveness, barriers to engagement and the extent to which students feel social, psychological and environmental factors either facilitate and/or impede help-seeking. A qualitative study was undertaken to obtain a subjective view of the experiences of participants using semi-structured interviews and data analysis employing IPA.

### Research design

A qualitative research method was used to investigate the nuanced, multilayered experiences of students engaging with digital mental health resources. The qualitative approach is especially appropriate for capturing the lived experience of individuals and provide a deep and layered understanding of personal meanings and perspectives (Smith and Osborn, 2014). Through an IPA approach, the present study aimed to gain insight into how participants of this study make sense of their experiences with internet-based, digitally-supported mental health tools. Because the interviews focused on students’ personal experiences and perspectives on digital mental health resources, and the students who participated came from different universities, reliable cross-campus service data were not consistent across institutions. Therefore, this study did not systematically assess or compare the mental health support infrastructure of institutions.

### Sampling approach

Chinese university students represent a large, highly digitalized population (Xiang et al., 2018). However, research on how this group uses digital mental health resources remains limited, despite a substantial gap between mental health needs and actual help-seeking or treatment in China (Ministry of Education of the People’s Republic of China, 2024; China Internet Network Information Center [CNNIC], 2025). To ensure relevance to the research aims, participants were eligible if they (1) were current university students in China, (2) had used at least one digital mental health resource within the past six months and (3) provided informed consent. Consistent with the idiographic focus of IPA, purposive sampling was used to recruit information-rich cases with direct, recent experience of digital mental health resources (Creswell, 1994). Recruitment began with student leaders assisting in identifying students who had used digital mental health resources and were willing to participate in interviews. After initial contact, seven students expressed interest. We then used snowball sampling, having these seven interested students recommend peers in their social networks who had also used digital mental health resources. Ultimately, 18 students were contacted; 10 agreed and completed the interview (5 men and 5 women), while 8 students declined to participate or did not respond after the initial contact. We did not exert any follow-up pressure on those who did not respond. Because participation depends on an individual’s willingness to share their experiences, the sample may overrepresent those students who have experienced more profound or emotionally intense experiences with digital mental health tools.

In line with IPA, the study adopted a small, relatively homogeneous sample to prioritize depth, analytic richness and meaning-making rather than statistical representativeness; transferability is therefore supported through thick description and readers’ assessment of contextual resonance with similar educational, cultural and digital mental health settings.

### Data collection

The data were gathered using semi-structured interviews, which are a particularly suitable method in qualitative research when examining a complex phenomenon in a flexible yet focused way (Kallio et al., 2016). Semi-structured interviews enable investigators to pose open questions, while permitting participants to expand on the responses and into different areas of concern. The interview guide was derived from the research questions and the theoretical framework of the study and included areas relating to participants’ motivations for using digital mental health, perceived effectiveness, barriers they experienced and social and psychological factors that contributed to their help-seeking.

The interviews were held one-on-one over Zoom, Google Meet or Skype – based on the preference of the respondent. The time spent on each interview was around 45–60 min. The interviews were recorded with the participants’ permission in order to allow verbatim transcription, from which data were subsequently analyzed. The interview questioning was developed in a way that stimulated participants to think more deeply about their own lived experiences and perspectives regarding digital mental health technology. Some of the interview questions were:

Tell me about using digital mental health tools.

What prompted you to look outside traditional sources of assistance?

Now what are some barriers or things that were hard for you to get those resources?

Do digital mental health tools make you uncomfortable because of their anonymity?

What part, if any, did social factors like friends, family or support groups play in your decision to use digital mental health tools?

The interviews took place in a private environment where the participants felt relaxed to be able to express their thoughts and feelings. At the end of the interview, an appreciation for the time spent was expressed to participants, and they had the possibility to ask questions or receive further information on the study. Ethical considerations were also taken into account to secure participants’ rights in this study’s data gathering.

### Data analysis

Data were analyzed using IPA following Smith (2016). Interviews were transcribed verbatim, de-identified and analyzed idiographically. TAM and SDT were used as the reference framework to identify the keywords, coding and sub-theme. During analysis, transcripts were read and reread, and preliminary descriptive, linguistic and conceptual notes were developed prior to identifying emergent themes. Themes were then iteratively refined using an inductive approach, with ongoing cross-case comparison to enhance coherence while preserving idiographic depth.

Two researchers independently analyzed the interview data and developed initial coding and theme structures. Differences were discussed and resolved through consensus, and analytic decisions were documented to support transparency and dependability. Reflexivity was maintained throughout the study: researchers engaged in continuous reflexive memo-writing to interrogate assumptions related to digital mental health and help-seeking and to monitor how these assumptions might shape interpretation. An audit trail was maintained across the analytic steps, and the final write-up integrates interpretative commentary with verbatim extracts to demonstrate grounding in participants’ accounts. Reflexivity was embedded throughout the analytic process. Both researchers maintained reflexive memos to document assumptions and evolving interpretations related to digital mental health and help-seeking, and they revisited these reflections through repeated analytic meetings to examine how their perspectives might shape coding and theme development. Decisions arising from these discussions were recorded in an audit trail, and the final report presents themes with verbatim extracts alongside interpretative commentary to ensure transparency and grounding in participants’ accounts.

### Ethical considerations

This study adhered to established ethical standards for research involving human participants. Written informed consent was obtained prior to participation, and participants were informed of the study purpose, procedures and potential risks, as well as their right to refuse or withdraw at any time without penalty. Participation was entirely voluntary, and no financial or material compensation was provided.

Online interviews were used based on participants’ preference and feasibility because participants were recruited from different universities. Prior to each interview, participants were advised to choose a private setting to reduce the risk of being overheard, and the potential privacy limitations of online platforms were clearly explained. Although the risks of participation are minimal, participants are informed that they can access mental health support services in case any discussion causes discomfort or emotional distress.

To protect confidentiality and privacy, participants were assigned pseudonyms and identifying information was removed from transcripts. Audio recordings and transcripts were stored in password-protected files, with access restricted to the research team, and all data were handled and reported in a manner that prevented identification of individuals or institutions. The authors declare no conflicts of interest.

## Findings

The findings are presented in two sections: (1) sociodemographic profile of participants and (2) thematic analysis.

### Sociodemographic and mental health context of participants

Table 1 represents the study sample consisted of ten university students, with an equal gender distribution: 50% male (*n* = 5) and 50% female (*n* = 5). Participants ranged in age from 23 to 30 years, with the majority (70%, *n* = 7) aged between 23 and 30 years, indicating a predominantly postgraduate student population. The age range of participants (23–30 years) indicates that the sample largely comprised postgraduate students. Undergraduate students aged 18–22 were underrepresented, which may have influenced the nature of reported help-seeking behaviors and engagement with digital mental health tools.

**Table 1.** Sociodemographic and mental health context of participants Table 1. long description.

Participant ID	Gender	Age	Program of study	Digital mental health resource used	Reason for use	Mental health status
P1	Male	23	Engineering	Mobile app (Yi Xinli/Linjian Liaoyushi)/Chatbot (Doubao)	Academic stress, anxiety symptoms	Moderate distress
P2	Male	24	Business	AI-driven chatbot/ Mobile app (Chao Xi)	Emotional stress, stigma-related hesitation	Mild to moderate distress
P3	Female	25	Social sciences	Mobile app /Online counseling (Jiandan Xinli)	Anxiety, emotional overwhelm	Acute distress
P4	Female	26	Business	Mobile app (Chao Xi)	Stress, low mood, digital fatigue	Mild distress
P5	Male	27	Engineering	AI-driven chatbot/ Mobile app (Yangxin Xinli)	Anxiety, nighttime emotional distress	Moderate to acute distress
P6	Female	28	Engineering	Mobile app (Wu Zhihong Xinli)	Emotional exhaustion, burnout	Moderate distress
P7	Male	29	Social sciences	Mobile app/Online counseling (Jiandan Xinli)	Persistent stress, self-reliance concerns	Moderate distress
P8	Female	30	Social sciences	Mobile app (Yi Xinli)	Anxiety, cognitive overload	Mild to moderate distress
P9	Male	23	Business	AI-driven chatbot (Only)	Stress, frustration with impersonal responses	Mild distress
P10	Female	24	Engineering	Mobile app /Online counseling (Jiandan Xinli)	Anxiety, need for continuity of care	Moderate distress

*Note:* Participants were not asked to disclose formal psychiatric diagnoses. Reason for use reflect participants’ own descriptions of stress, anxiety, emotional distress, burnout or adjustment difficulties. Mental health status represents participants’ perceived level of distress at the time of engaging with digital mental health resources.

In terms of academic discipline, 40% (*n* = 4) of participants were enrolled in engineering programs, while 30% (*n* = 3) were studying business-related courses and 30% (*n* = 3) were from the social sciences. This distribution reflects moderate disciplinary diversity within the sample.

Regarding engagement with digital mental health resources, 40% (*n* = 4) of participants primarily used mobile mental health applications, 30% (*n* = 3) engaged with AI-driven chatbots and 30% (*n* = 3) accessed online counseling services. This indicates relatively balanced exposure across different forms of digital mental health support.

With respect to self-reported mental health concerns, 100% of participants (*n* = 10) described experiencing some form of psychological distress, most commonly academic stress, anxiety symptoms, emotional exhaustion or burnout. These concerns were self-identified through participants’ narratives rather than clinical assessment.

Prior engagement with in-person counseling services was reported by 40% of participants (*n* = 4), while 60% (*n* = 6) indicated no previous face-to-face counseling experience, suggesting that for a majority of participants, digital mental health tools functioned as a primary or initial avenue for help-seeking.

Participants’ perceived mental health status at the time of using digital resources varied. 30% (*n* = 3) described their distress as mild, 40% (*n* = 4) reported moderate distress and 30% (*n* = 3) indicated moderate-to-acute or acute levels of distress. This variation highlights heterogeneity in psychological readiness and need at the point of digital engagement.

Overall, while the sample was demographically homogeneous in terms of age range and educational status, meaningful variation was observed in psychological context, prior service use and perceived distress levels. This diversity contributed to analytic richness and supported idiographic and comparative interpretation within the IPA framework.

### Thematic analysis

The data analysis revealed four key themes: (1) Accessibility and convenience; (2) Perceived effectiveness of digital interventions; (3) Barriers to engagement and (4) Psychological influence on help-seeking behavior. Each theme represents significant aspects of the participants’ experiences and perceptions related to digital mental health resources.

#### Accessibility and convenience

The significance of access and utility was a consistent finding from the analysis of the lived experience of digital mental health tools for a student population. Participant accounts generated evidence for the benefits and challenges of the accessibility and flexibility of digital platforms. Participants emphasized that the large removal of the traditional barriers to mental health care was imperative. The main barriers for the participants before entering the digital platforms were economic barriers, the long waiting time and the distance to the counseling centers. For example, P3 reflected; *“I could have a session with a mental health coach in minutes literally, and that release of being able to have immediate access to support, is tremendous.”* This narrative demonstrates how digital resources overcame logistical constraints that allowed students to reach out in their own time, when they felt they most needed the help.

A critical aspect that came up during the interviews was the availability of digital mental health tools around the clock. P5 expressed enjoying the freedom and security of being able to use online mental health support at night when they could not access in-person care. “*I feel so terribly overwhelmed at night … I appreciate having something like online mental health chatbots that I can turn to like this,”* P5 expressed. This underscores the importance of being able to intervene while students are most at risk, a function that digital platforms can provide. Participants also commonly referred to the issue of anonymity. Students like P2, who were embarrassed to seek in-person help, found that digital spaces provided a safe environment devoid of judgment where their issues could be dealt with, *“I didn’t want my classmates to gossip about my struggle, but when I contacted it through an online (source) it really helped.”*

Despite its advantages, however, depersonalization was a challenge for some users of digital mental health. P4 explained the dissonance they experienced while interacting with computer-mediated AI chatbots by saying, *“I don’t feel like someone on the other side feels my pain. And it was a sentiment expressed by others, many who complained about the bland answers they got.”* This was the feeling of others as well who were disappointed with the bland reply they also received. These participants yearned for human touch and care that is difficult to replicate through digital means. P6 also observed that having a digital platform for support was very useful at first, but “keeping engagement up became hard” without the physical support and care in in-person therapy, *“There was no one to hold me accountable like a real therapist.”* The lack of personalized engagement seemed to be a key challenge to some participants in terms of the sustainable use of digital mental health resources.

#### Perceived effectiveness of digital interventions

The perceived effectiveness theme was identified as a key factor for understanding students’ experiences of digital mental health interventions. The experiences of university students showed the intricate relationship between their beliefs about the beneficial effects of digital interventions and their drawbacks in meeting psychological needs in their entirety. Both positive and negative perceptions of the usefulness of the digital resources were reported by students, and it is evident that (mis)perceptions of utility influenced the help-seeking and usage behavior of the students on the online platforms.

Participants also cited the role of digital interventions in learning how to self-regulate (e.g., mindfulness, cognitive restructuring, stress management). P8 reported about their experience with an AI-based CBT app and held that, *“I had like my personal coach to reframe my thinking when I was spiraling down.”* This narrative demonstrates that the digital tools gave participants practical coping strategies and emotional insights to help them manage their mental health struggles on their own. These resources seemed useful in particular for students that preferred self-guided methods for engaging with mental health, as the apps allowed students autonomy in working to support their affective needs. Likewise, P3 also noted that the digital resource taught him *to “learn practical skills that helped me to manage my anxiousness,”* highlighting students’ empowerment towards learning skills to regulate their emotions.

Participants also conceded that digital tools are valuable, but they saw them as adjunctive rather than replacement interventions for traditional therapy. P10 reflected, *“My go-to strategy is to see a regular therapist and use the online resources in between.”* This participant’s experience is typical of a moderate user, where digital interventions were used in addition to face-to-face therapy and as a means of continuing contact with mental health support in between scheduled appointments. Other participants, such as P5, articulated frustrations when their universities promoted digital mental health platforms as the solution, “*Sometimes it kind of felt like the university didn’t really want to reach out to hire better counselors, because they wanted everyone just to have mental health app.”* This feeling implies that while digital programs can give helpful backing, students appreciate the personal dimension and depth offered by human therapists and thus digital tools were insufficient to serve alone. The idea of digital interventions as adjunctive highlights the tension between what students want and what many university-led digital platforms provide which is not able to address all emotional and psychological needs.

Although there are advantages of digital interventions, some participants expressed annoyance at receiving impersonal responses as the messages they receive are generic. P9 described submitting their deeply personal narrative to a mental health platform, but receiving *“a pre-written automated response that wasn’t even entirely relevant to what I said.”* This reflected an emerging theme that digital interventions did not always offer the personalized support that students wanted. P6 described, *“I like what I used to begin with but then wasn’t much helpful because I feel that I am just another inclusion to their database.”* These narratives may indicate that a lack of personalization in digital interventions can contribute to the experience of feeling distant and disconnected from a program, and as a result reduce perceived program effectiveness. Participants stated that the lack of human understanding in the digital world made them feel not understood and disengaged from the supportive environment, which influenced their future use of these tools.

#### Barriers to engagement

The theme of barriers to engagement was a continued difficulty when considering the use of digital mental health resources among students at a university. The experiences of the participants illuminated a number of barriers which prevented them from using those digital tools – for their use over indefinite time – more intensively. Although digital tools were both easy to access and convenient, few perceived them as credible and a number of barriers, including psychological ones, prevented students from using them on a regular basis.

Among the most salient barriers to engagement was the participants’ disbelief in the usefulness of digital interventions for mental health, and a lot have doubts that digital platforms can actually get at the real emotional and psychological pain. For example, P2 reflected, “*The advertisements I had seen about mental health apps say they can ‘change my mindset,’ but it felt really impractical.”* P5 echoed this sentiment: “*Apps help me calm down temporarily, but they don’t solve real (deep) problems like anxiety.”* This lack of trust betrays an underlying fear that digital programs are not enough in the long run and that they might not provide the level of care required for anything more serious than a transitory episode of mental unwellness. The participants appeared to question whether digital materials would endure and were less convinced that they might be ultimately be a substitute for human-instigated therapy – the way these apps are initially tested and compared. Such uncertainty acted as a barrier for continued use, since students were skeptical that the amount of effort and time that they spent on digital platforms would translate into any actual or sustained improvement in their mental health.

Digital fatigue, which is a term used by participants to indicate fatigue due to the overuse of technology, was another key barrier encountered by participants. P6, for example, noted, *“I just don’t want to be on my computer or my phone all day after I get back from class. The last thing I want to do is open another app.”* This narrative also illustrates the challenge of balancing academic, personal and mental well-being in a highly digitized world. Given the overabundance of screen time people already endure on a regular basis, participants said they were overwhelmed by the prospect of using a new app to take care of their mental health. Participants also mentioned issues with the usability of apps, including apps that were hard to use and apps that had complex interfaces. P6 was annoyed about the appearance of one of the mental health apps, regarding it being *“not very nice to look at,” “dull and was quite restrictive to use.”* Usability issues such as these impacted on participants’ willingness to use digital mental health tools in this context, as clunky or difficult platforms had a negative impact on the user experience and resulted in disengagement.

#### Psychological influence on help-seeking behavior

The theme of psychological influence was identified as a key issue in relation to how participants manage the use of digital mental health resources to seek support. Participants illuminated that it is their complex psychological landscape that determines their readiness to seek help and use digital interventions. These psychological factors, which included self-perception, internal resistance, social stigma as well as peer influence, were critical in shaping decisions on whether to seek support from digital resources or not.

An important psychological factor that affected help-seeking behavior was participants’ self-perception and psychological readiness to accept their mental health problem. For some participants, it was especially hard to admit they needed help. P6 stated, *“I’ve worked with people coming to me for them in family within my work, and I kind of felt I’d be able to help however for me to accept that I needed support even to an app it was quite un-nerving me.”* This narrative also highlights how being seen emotional tough or self-sufficient can act as a barrier to seeking help. People who were less likely to use digital tools for support were those who believed they could control their emotions on their own. The intersensory paradox of feeling self-supportive while feeling helpless was a formidable psychological barrier to help-seeking for mental health resources. This self-resistance was high among those who felt that asking for help would be considered weak or inept at handling their own issue. Perceived fear of judgment and stigma of using digital mental health tools were also important psychological reasons for students not engaging with digital mental health. Participants were afraid that people would think they were “weak” or “incompetent” if they discovered they’d gone for help. It was illustrated by P5 who commented, “*I know it’s just an app but I feel like I can’t handle my stuff. I realize that’s a shaming thought to many readers, but it’s what I actually feel.”* The fear of being judged, especially by peers or potential employers, acted as a psychological barrier preventing the participants from getting the fullest benefit from digital support.

Peer influence and social norm were important determinants of help-seeking behaviors. Participants reported that social network and peer attitudes towards mental health led to their decisions to use digital mental health resources. P3 added; *“I have a good friend who told me their experience with a mental health app. With out that conversation, I would have never been even remotely comfortable in discussing the potential of technology-based support options.”* This is an example of the peer-sensitivity that promotes the normalization of mental health help-seeking. For some participants, watching peers be open about their use of digital mental health tools helped them to feel more comfortable doing the same. Yet in contrast, P7 revealed that among their peer network, supporting others with mental health problems was often dismissed as unnecessary practice. He reflected, *“Some of my friends from coaching thought mental health apps were pointless… ‘Go outside and walk barefoot, touch the grass, or interact with nature,’.”* In these settings, participants discouraged each other from seeking help and were embarrassed to disclose they had used digital mental health platforms due to potential social invalidation. The influence of social validation and peer reinforcement on help-seeking behaviors draws attention to the part that external influence plays in whether or not students are comfortable with the idea of using digital resources.

#### Safety, accountability and escalation concerns

The participants’ narratives revealed a profound tension between the immediate accessibility of digital platforms and deep-seated anxieties regarding clinical safety, professional accountability and the systemic mechanisms for crisis escalation. Many users leveraged digital support as a critical “bridge” during after-hours crises when traditional services were inaccessible; as P5 noted, “*I feel so terribly overwhelmed at night… I appreciate having something like online mental health chatbots.*” However, this convenience was frequently offset by the perceived depersonalization of automated interventions. For instance, P9 expressed frustration with “*pre-written automated responses that weren’t even entirely relevant*,” suggesting that generic AI-driven feedback failed to meet the specific emotional nuances of their distress.

Furthermore, a central concern was the absence of a clear accountability mechanism, which is a hallmark of the traditional therapeutic alliance. P6 highlighted this void, remarking that “*there was no one to hold me accountable like a real therapist.*” This perceived lack of human oversight led to skepticism regarding institutional motives, with some participants viewing university-promoted apps as a “*cheap substitute*” for robust counseling provision rather than a genuine supplement. This sentiment was poignantly captured by P5, who observed that “*it felt like they wanted everyone just to have a mental health app*,” reflecting a deep-seated resistance to the “*app-ification*” of mental health care that prioritizes technical scalability over the ethical safeguards and human trust essential for safe intervention.

## Discussion

The current study examined university students’ use of digital mental health services, with an emphasis on how these platforms shape help-seeking behaviors. Drawing on semi-structured interviews with ten participants, the findings highlight how accessibility and convenience, perceived effectiveness, barriers to engagement and key psychological factors jointly influence students’ engagement with digital mental health resources. To further interpret these patterns and clarify potential mechanisms, the TAM and SDT were used as reference frameworks to inform the identification of key terms, coding and the organization of sub-themes. Specifically, TAM helps explain initial uptake through PEOU and PU, whereas SDT offers a complementary lens for understanding how autonomy, competence and relatedness needs shape motivation and sustained engagement.

### Accessibility, convenience and personalization tensions

Participants strongly valued the access and flexibility offered by digital mental health resources. This aligns with evidence that digital services, including online therapy, can reduce traditional barriers to care (Bucci et al., 2019; Anser et al., 2025). However, convenience alone was insufficient. Several participants described AI-driven chatbot responses or automated support as formulaic and impersonal, echoing prior work showing that digital solutions may struggle to replicate the emotional connection present in face-to-face therapy (Montagni et al., 2019; MacDonald et al., 2024). Together, these accounts underscore a core tension between convenience and the need for individualized, user-centric support and illustrate how PEOU can function as a primary driver of initial adoption while still leaving critical needs unmet.

### Digital mental health as adjunctive support

A further theme that emerged concerned the perceived reliability of digital mental health tools. Participants generally viewed digital interventions as ancillary rather than a substitute for usual care. Some found CBT apps and mindfulness programs helpful for managing stress and anxiety independently, consistent with evidence for digital platforms in stress management (Harrer et al., 2018). At the same time, participants raised concerns about limited lasting impact. Tools were often perceived to offer short-term relief while failing to address deeper or more complex contributors to distress. These reflections are consistent with broader concerns about the sustainability and scope of digital interventions, especially for individuals experiencing more severe difficulties (Ebert et al., 2017). Overall, these findings reinforce the view that digital mental health interventions are most appropriately positioned as add-on treatments to standard care, with PU under TAM contingent upon personalization and relevance to individual needs.

### Barriers to engagement and digital fatigue

Despite advantages in accessibility, digital mental health resources also presented notable engagement challenges. Many participants expressed skepticism about whether digital tools were capable of meaningfully addressing complex emotional experiences. Similar doubts have been documented by Torous et al. (2025), who noted persistent uncertainty regarding the depth of assistance digital platforms can offer.

Digital fatigue emerged as an additional barrier, particularly in the context of extensive daily screen exposure related to academic and social activities. Participants described reluctance to engage further with digital tools due to exhaustion from prolonged technology use, a concern consistent with Spytska (2024), who identified digital fatigue as an increasing challenge in technology-assisted interventions. Usability issues, including poor design and unresponsive interfaces, further contributed to disengagement. Failures to satisfy relatedness needs, as described by SDT, appeared to exacerbate fatigue and erode trust in digital platforms.

### Psychological and social influences on help-seeking

Psychological factors played a significant role in shaping help-seeking attitudes. Perceptions of self-sufficiency and resilience were frequently cited as barriers to seeking support through digital mental health tools. Participants described internal resistance to help-seeking rooted in fear of appearing weak, a phenomenon supported by stigma literature identifying perceived incompetence as a key barrier to help-seeking (Corrigan and Watson, 2002).

Stigma and concern about social judgment further constrained engagement, consistent with findings that shame and stigma impede professional help-seeking (Shahwan et al., 2020). Conversely, peer influence exerted a facilitating effect when positive experiences were shared, reinforcing the role of social support networks in normalizing help-seeking and reducing stigma (Henderson et al., 2013). Together, these findings suggest that autonomy and competence shaped readiness to engage with digital tools, while stigma and self-reliance moderated help-seeking behavior.

### Gender, cultural context and selection bias

Gendered and culturally shaped norms may help explain why some participants preferred low-disclosure, self-guided digital support while simultaneously expressing skepticism toward sustained use. Prior evidence suggests that women tend to report more positive attitudes toward professional psychological help than men, often linked to gender-role expectations around emotional disclosure and self-reliance (Qiu et al., 2024). Within Chinese university contexts, qualitative work similarly highlights how stigma, concerns about social judgment, and cultural expectations can discourage formal help-seeking and increase reliance on more private forms of support (Ning et al., 2022). Selection processes further condition interpretation. Because recruitment relied on voluntary participation and opportunistic snowball sampling, perspectives from disengaged or skeptical users may be underrepresented (Ting et al., 2025). Together, these lenses suggest that “acceptance” of digital mental health tools reflects not only usability and perceived benefit but also socially patterned disclosure norms and recruitment-related sampling constraints, which should be considered when assessing transferability (Torous et al., 2025).

### Ethical and governance implications of AI-mediated support

Participants generally viewed digital mental health tools as useful adjuncts, but their accounts also raised governance-relevant risks. Students reported varying levels of distress and some relied on digital support during nighttime emotional escalation when in-person services were unavailable, making perceived limits in emotional attunement and accountability more consequential. These concerns resonate with emerging evidence on AI-mediated mental health risks, including emotional dependency, inappropriate responses for vulnerable users, inadequate crisis detection and failures in timely suicide referral (Zidaru et al., 2021; Hui et al., 2022; Lei, 2025).

Participants described AI interactions as lacking “human presence,” reported discouragement when replies felt generic or irrelevant and questioned responsibility when university-promoted apps appeared to substitute for counseling rather than complement it. Together, these findings suggest that ethical deployment requires governance beyond technical performance, foregrounding responsibility, escalation and oversight. Considering these, three priorities follow for university implementation. Digital tools should be embedded within stepped-care pathways with explicit crisis escalation options (Brennenstuhl et al., 2025). Governance should ensure transparency about system capabilities and limits, safeguards against overdependence and accountability mechanisms, with human oversight sustaining trust (APA Health Advisory on the Use of Generative AI Chatbots and Wellness Applications for Mental Health, n.d.). Digital mental health literacy should also be strengthened so students can judge tool credibility, set appropriate boundaries and recognize when professional or emergency help is needed (Tian et al., 2024). These implications align with current debates cautioning against uncritical deployment of AI-mediated tools in contexts where cultural norms and institutional constraints shape help-seeking (Ali et al., 2025; Gazit and Gazit, 2025).

## Limitation

This study has several limitations that should be considered when interpreting the findings. The small sample size (*n* = 10) limits experiential breadth and reflects the idiographic orientation of IPA, which prioritizes depth over generalizability. All participants were recruited from universities in China, and sociocultural norms surrounding mental health, stigma and self-reliance may shape engagement in ways that differ from other educational contexts. The age range of participants (23–30 years) indicates a predominance of postgraduate students, with undergraduate students underrepresented; academic stage may influence autonomy, digital literacy and help-seeking behaviors, and therefore the findings may not fully reflect the experiences of younger university populations. Additionally, the absence of detailed information on diagnoses, symptom severity, treatment history and socioeconomic status constrains interpretation of how clinical and structural factors shape help-seeking. Finally, institutional variation was not systematically examined. Differences across universities in counseling service capacity, the integration and promotion of digital tools and the clarity of referral and crisis response pathways may have influenced participants’ experiences and expectations, making it difficult to disentangle individual perceptions from campus-level conditions. Taken together, these limitations suggest that the findings provide context-specific insights into the experiences of predominantly postgraduate students within Chinese universities and highlight the need for future research incorporating broader age ranges, stronger undergraduate representation, richer clinical and socioeconomic context and comparative designs across institutions to enhance cross-contextual understanding.

## Conclusion

This study examined how university students engage with digital mental health resources and how these experiences shape help-seeking. These exploratory insights that may inform similar university students’ digital mental health help-seeking. The findings offer preliminary, context-specific insights into how university students interpret and engage with digital mental health resources, highlighting areas for future research and cautious application in similar contexts. Participants valued accessibility, convenience and anonymity, which lowered the threshold for initial use, especially for those reluctant to seek face-to-face help. However, many also reported dissatisfaction with generic or impersonal responses, indicating that sustained engagement depends on more user-centered and emotionally responsive support. Overall, students viewed digital tools as adjuncts rather than substitutes for professional mental health help. While some resources helped with self-management skills such as stress regulation and cognitive reframing, participants questioned their long-term effectiveness, particularly for more severe difficulties, supporting the need for blended pathways that link digital use with timely human care. Help-seeking was further shaped by stigma, self-reliance and peer norms, with peer endorsement appearing to reduce reluctance and increase comfort. Finally, although digital resources may improve access and continuity, ethical deployment requires human oversight, clear governance and safeguards to protect vulnerable users.

## Data Availability

The original contributions presented in this study are included in the article/supplementary material. Further inquiries can be directed to the corresponding author(s).

## Table 1. Long description

The table has seven columns: Participant I D, Gender, Age, Program of study, Digital mental health resource used, Reason for use, and Mental health status. From top to bottom, the rows are as follows. P1: Male, 23, Engineering, Mobile app Yi Xinli or Linjian Liaoyushi and chatbot Doubao, reason is academic stress and anxiety symptoms, moderate distress. P2: Male, 24, Business, A I-driven chatbot and mobile app Chao Xi, emotional stress and stigma-related hesitation, mild to moderate distress. P3: Female, 25, Social sciences, mobile app or online counseling Jiandan Xinli, anxiety and emotional overwhelm, acute distress. P4: Female, 26, Business, mobile app Chao Xi, stress, low mood, digital fatigue, mild distress. P5: Male, 27, Engineering, A I-driven chatbot and mobile app Yangxin Xinli, anxiety and nighttime emotional distress, moderate to acute distress. P6: Female, 28, Engineering, mobile app Wu Zhihong Xinli, emotional exhaustion and burnout, moderate distress. P7: Male, 29, Social sciences, mobile app or online counseling Jiandan Xinli, persistent stress and self-reliance concerns, moderate distress. P8: Female, 30, Social sciences, mobile app Yi Xinli, anxiety and cognitive overload, mild to moderate distress. P9: Male, 23, Business, A I-driven chatbot Only, stress and frustration with impersonal responses, mild distress. P10: Female, 24, Engineering, mobile app or online counseling Jiandan Xinli, anxiety and need for continuity of care, moderate distress. The table note states that participants were not asked to disclose formal psychiatric diagnoses; reasons for use reflect participants’ own descriptions, and mental health status is self-perceived at the time of using digital resources.

Navigate back to Table 1..
