# Supplementary material for: The role of digital mental health resources in encouraging help-seeking: A qualitative study of university students’ experiences
Source: Glob Ment Health (Camb). 2026 May 18;13:e116. doi: 10.1017/gmh.2026.10216 (PMC13279960; doi:10.1017/gmh.2026.10216)
Supplement: Peer review history

# Reviewer report: Review: The role of digital mental health resources in encouraging help-seeking: A qualitative study of university students’ experiences — R0/PR2

**DOI:** 10.1017/gmh.2026.10216.pr2

**Authors:** Swasti Mishra

This qualitative study examines the lived experiences of 10 Chinese university students engaging with digital mental health resources through semi-structured interviews and Interpretative Phenomenological Analysis (IPA). While the research addresses a timely and important topic in digital mental health, the manuscript requires substantial revisions to meet publication standards. The study identifies key themes around accessibility, perceived effectiveness, barriers to engagement, and psychological influences on help-seeking behavior. However, significant methodological concerns, presentation issues, and analytical gaps must be addressed before acceptance.

Recommendation: Major Revision

1. Sample Size and Generalizability

• A sample of 10 participants is extremely small for any qualitative study, even for IPA. While IPA can work with smaller samples, 10 is at the lower acceptable limit, and this severely constrains the richness and transferability of findings.

• All participants are from Chinese universities—this geographic and cultural specificity is not adequately acknowledged or discussed in terms of generalizability to other educational contexts.

• The abstract claims findings are ‘relevant for digital interventions used in mental health support,’ but this is an overstatement given the limited sample.

Recommendation: Either expand the sample to at least 15-20 participants for greater robustness, or significantly temper claims about generalizability and position the study as preliminary/exploratory. Clearly state limitations in the abstract and conclusions.

2. Insufficient Sampling Detail and Recruitment Bias

• The paper states recruitment was ‘opportunistic’ using snowball sampling, which introduces significant selection bias. Participants self-selected because they were willing to discuss their experiences, potentially overrepresenting those with strong opinions (positive or negative) about digital tools.

• No information is provided about recruitment response rates, non-response rates, or whether participants compensated (though ethical approval mentions compensation requirements for some studies).

• No details on how many participants were contacted or approached before the final 10 agreed to participate.

• The justification that purposive sampling ‘provides the range of diversity that represents the highest quality of thick description’ (citing Creswell 1994) is circular reasoning without demonstrating actual diversity achieved beyond the demographic table.

Recommendation: Provide explicit details on recruitment procedures, response rates, and acknowledge selection bias. Consider using quota sampling to ensure greater demographic representation.

3. Lack of Participant Diversity

• All 10 participants are current students aged 23-30, which is quite narrow for ‘university students.’ Most universities have students from age 18 onwards. This age range skews toward postgraduate students.

• No information on undergraduate vs. postgraduate status, which may significantly affect help-seeking behaviors and technology adoption.

• No information on mental health status of participants (whether they have diagnosed conditions, severity, duration of symptoms), which is fundamental context for understanding help-seeking.

• No information on previous mental health treatment history (whether participants have used traditional therapy before).

Recommendation: Provide additional demographic variables (year of study, prior mental health history, SES indicators) and explicitly discuss how these may limit findings.

MINOR CONCERNS

1. References formatting: Some references are incomplete or improperly formatted (e.g., missing page numbers).

2. Citation accuracy: Verify all citations are correctly attributed. For example, Davis (1989) on TAM is correctly cited, but ensure all recent citations (2024, 2025) are actually published or in press.

3. Keyword relevance: ‘Assessment tools’ and ‘Depersonalization’ (from the abstract metadata) don’t appear to be major themes in the paper. These keywords should be revised to better match the actual content (e.g., ‘help-seeking behavior,’ ‘peer support,’ ‘digital fatigue’).

4. Institutional variation: While the ethical approval is noted (SUCVC-2024-008), no information about whether universities differed in their mental health support infrastructure, which could affect participant experiences.

# Reviewer report: Review: The role of digital mental health resources in encouraging help-seeking: A qualitative study of university students’ experiences — R0/PR3

**DOI:** 10.1017/gmh.2026.10216.pr3

The qualitative investigation into university students’ experiences with digital mental health tools is timely because of the proliferation of AI tools. The study identifies themes around accessibility, perceived effectiveness, barriers to engagement, and psychological influences on help‑seeking. The use of Interpretative Phenomenological Analysis is used for the research aims, and the findings contribute meaningfully to understanding how young adults navigate digital mental health support.

Comments:

1. There could be stronger theoretical integration and deeper engagement with emerging risks associated with AI‑mediated mental health support. Recent evidence highlights significant safety concerns—including emotional dependency, AI‑induced psychosis, harm‑related responses, and failures in suicide‑referral protocols—that are directly relevant to the study’s conclusions about digital tools as adjuncts to care. Clarify the implications for ethical deployment and governance.

2. The sample size and cultural specificity are appropriate for IPA but limit transferability. There could be more reflection on gendered help‑seeking patterns, cultural context, and potential selection bias. Revisions are needed to enhance conceptual depth and situate the findings within current debates on digital mental health safety and oversight.

3. Theoretical frameworks are underutilized. TAM and SDT are introduced but not fully integrated into the analysis. Clearer mapping of themes to theoretical constructs would strengthen coherence.

4. Implications for practice and governance need expansion.

The manuscript should address crisis‑response limitations, ethical safeguards, digital literacy, and the need for human oversight in digital mental health interventions.

5. Sample limitations require deeper reflection.

Cultural specificity, digital literacy differences, and potential selection bias should be more explicitly acknowledged in the limitations section.

Minor comments:

6. Clarify key terms such as “digital fatigue” and “psychological readiness.”

7. Improve flow in the introduction to better connect the rationale and research gap.

8. Provide additional detail on reflexivity and coding procedures.

9. Correct minor typographical errors throughout the manuscript.

10. Expand ethical considerations related to online interviews and digital mental health contexts.

# Editor report: Recommendation: The role of digital mental health resources in encouraging help-seeking: A qualitative study of university students’ experiences — R0/PR4

**DOI:** 10.1017/gmh.2026.10216.pr4

**Affiliations:** Department of Primary Care & Mental Health , University of Liverpool, UK, United Kingdom of Great Britain and Northern Ireland

Dear Authors 

We have now received reviewer comments on your manuscript. based on the comments, we recommend major revisions to the current draft for it to be considered suitable for publication. 

Regards

Siham

# Editor report: Decision: The role of digital mental health resources in encouraging help-seeking: A qualitative study of university students’ experiences — R0/PR5

**DOI:** 10.1017/gmh.2026.10216.pr5

**Affiliations:** London School of Hygiene & Tropical Medicine, United Kingdom of Great Britain and Northern Ireland

No accompanying comment.

# Reviewer report: Review: The role of digital mental health resources in encouraging help-seeking: A qualitative study of university students’ experiences — R1/PR7

**DOI:** 10.1017/gmh.2026.10216.pr7

The revision addressed the concerns in a comprehensive and thoughtful manner. The authors have strengthened the manuscript’s theoretical integration, particularly by more explicitly linking participant experiences to relevant frameworks such as Self-Determination Theory (SDT) and the Technology Acceptance Model (TAM). Their addition of a dedicated discussion on emerging risks associated with AI-mediated mental health support—such as emotional dependency, crisis escalation limitations, and ethical governance—demonstrates a clear engagement with current debates and recent literature.

The reflection on sample limitations, including cultural specificity, gendered help-seeking patterns, and the potential for selection bias, has been deepened. The authors now situate their findings more clearly within the sociocultural context of Chinese higher education and acknowledge how these factors may influence transferability. The conceptual depth is enhanced by these additions and the new discussion on the broader implications for digital mental health safety and oversight.

The authors have also provided clearer mapping between emergent themes and theoretical constructs, thereby improving analytic coherence while maintaining fidelity to the interpretative phenomenological approach. The inclusion of a dedicated implications subsection, as well as expanded ethical considerations related to online interviews and digital mental health contexts, further strengthens the manuscript.

Minor issues, such as the operationalization of key terms, greater detail on reflexivity and coding procedures, and the correction of typographical errors, have been satisfactorily addressed in the revised manuscript.

Overall, the authors’ responses are satisfactory and substantially improve the rigor, clarity, and contextual transparency of the manuscript on digital mental health in education.

# Reviewer report: Review: The role of digital mental health resources in encouraging help-seeking: A qualitative study of university students’ experiences — R1/PR8

**DOI:** 10.1017/gmh.2026.10216.pr8

**Authors:** Swasti Mishra

I congratulate the author(s) for taking up the revision. The paper can be accepted in its current form.

# Editor report: Recommendation: The role of digital mental health resources in encouraging help-seeking: A qualitative study of university students’ experiences — R1/PR9

**DOI:** 10.1017/gmh.2026.10216.pr9

**Affiliations:** Department of Primary Care & Mental Health , University of Liverpool, UK, United Kingdom of Great Britain and Northern Ireland

Dear Authors 

We have received your revised manuscript and it has been accepted for publication. 

We will inform you of the next steps. 

Regards

Siham

# Editor report: Decision: The role of digital mental health resources in encouraging help-seeking: A qualitative study of university students’ experiences — R1/PR10

**DOI:** 10.1017/gmh.2026.10216.pr10

**Affiliations:** London School of Hygiene & Tropical Medicine, United Kingdom of Great Britain and Northern Ireland

No accompanying comment.
